# Cancer Survivors in Delaware: Impact of Comorbidity

**DOI:** 10.1192/j.eurpsy.2024.754

**Published:** 2024-08-27

**Authors:** S. Gupta

**Affiliations:** Delaware State University, Dover, United States

## Abstract

**Introduction:**

Delaware’s recent longevity and aging trends predict a continual increase in the number of cancer survivors. As the cancer survivors live longer and age, the prevalence of comorbid chronic conditions tends to increase. Dual burden of cancer and comorbid chronic conditions can have significant and wide-ranging ramifications for cancer survivors. Comorbidity potentially affects the development, stage at diagnosis, treatment options, recurrence and long-term survival of people with cancer. Detailed delineation of Delaware adult cancer survivors including an exploration of comorbidity is critical.

**Objectives:**

The primary objective was to characterize selected chronic conditions among Delaware adults with cancer in order to present: (i) disparities amongst cancer survivors by select sociodemographic and survivorship characteristics, and (ii) compare the prevalence of chronic conditions among cancer survivors and adult Delawareans without a cancer diagnosis.

**Methods:**

Combined data (2018, 2020 and 2021) for Delaware were obtained from the Behavioral Risk Factor Surveillance System. The final data set included 927 Delawareans with at least one type of cancer (excluding skin cancers other than melanoma) and 11,917 participants without a cancer diagnosis. Descriptive statistics examined sociodemographic characteristics and chronic conditions in Delawareans with and without a cancer diagnosis.

**Results:**

Amongst adult Delawareans, 5.1% (CI: 4.6–5.5) were cancer survivors. Across the state, the majority of cancer survivors (76.8%) reported having only one cancer diagnosis. In this sample of Delaware cancer survivors, 83.5% identified as White. Majority were female (57.4%), aged 65 or older (58.9%), had some college or more education (63.7%), and with an income of $50,000 or more (51.1%). Arthritis (46.3%), diabetes (21.5%), depression (18.7%), asthma (14.1%), chronic obstructive pulmonary disease (13.7%) angina (11.9%) and heart attack (11.6%) were the most prevalent comorbid conditions. Prevalence of certain chronic conditions was 2-3 times higher among cancer survivors. Nearly 23% reported not receiving instructions regarding cancer follow-up care.

**Conclusions:**

Cancer survivors have unique concerns. Results aim to facilitate targeted interventions aimed at coordinated managed care among cancer survivors in Delaware. This study bolsters the ongoing public health effort towards the Healthy People 2030 goal of increasing the proportion of cancer survivors.

**Disclosure of Interest:**

None Declared

